# Editorial of Harnessing the Power of T Cells: The Promising Hope for a Universal Influenza Vaccine

**DOI:** 10.3390/vaccines8030376

**Published:** 2020-07-11

**Authors:** Salman M. Toor, Varun Sasidharan Nair, Eyad Elkord

**Affiliations:** Cancer Research Center, Qatar Biomedical Research Institute (QBRI), Hamad Bin Khalifa University (HBKU), Qatar Foundation (QF), Doha 34110, Qatar; mstoor@hbku.edu.qa (S.M.T.); vsnair@hbku.edu.qa (V.S.N.)

The global burden of influenza-associated respiratory mortality is higher than previous estimates, with over 0.6 million deaths per year and additional mortality rates attributed to non-respiratory but influenza-related illnesses [1]. Numerous vaccines have been developed to protect against influenza infections. However, the efficacies of current influenza vaccines, which include cell-based vaccines, recombinant vaccines, inactivated influenza vaccine (IIV) and live attenuated influenza vaccine (LAIV) have dropped in recent years and do not provide long-term viral defense. Moreover, the hemagglutination inhibition (HAI) titers are widely considered to correlate with protection against influenza; however, patients with high titers have also been infected by influenza, while others without titers were protected [2]. Therefore, there is a real need to explore novel candidates of vaccine development and decrease mortality rates.

Clemens et al. have highlighted the role of influenza-specific T cell immunity in the development of a universal influenza vaccine [3]. The authors described the current arsenal of vaccines against influenza and emphasized on T cell-mediated antiviral immune responses. Importantly, the authors presented the different plausible approaches and challenges associated with the development of a universal broad-spectrum T cell vaccine for influenza. Other studies reported that unique antibodies restricted to the conserved hemagglutinin (HA) were found in humans and animals, prior to influenza exposure. These antibodies have broad specificity, including the HA clusters of influenza A and B (IAV and IBV), indicating a potential approach in the development of a universal influenza vaccine [4,5]. Of note, no further studies were reported for the development of such vaccines, revealing the difficulties in the physiological induction of the aforementioned antibodies. However, T cell- and antibody-mediated immune responses provide primary/broad level protection against influenza infections and the precise molecular mechanisms behind their protective roles remain unclear. This could be primarily due to the existence of heterosubtypic influenza viruses.

Current influenza vaccines are primarily based on strain-matched hemagglutination inhibition antibodies against variable surface glycoproteins, HA and neuraminidase (NA) [2]. T cells are at the forefront of naturally acquired protective antiviral immune responses and require activation via the antigen presentation of viral peptides. Importantly, peptide homology and conserved peptide sequences between different strains of influenza ensures their recognition and activation by memory T cells. T cell epitopes for vaccines are primarily derived from internal viral proteins, which are highly conserved [3]. Therefore, T cells elicit broader immunity against novel strains of influenza as they recognize the conserved internal viral components. Moreover, the presence of such T cell epitopes in influenzas A and B rationalizes the possibility of the development of a universal vaccine [3]. The role of cytotoxic T cells in initiating cross-reactive cellular immune responses and memory T cells in response to viral infections infers that the development of a successful T cell vaccine should initiate suitable CD4^+^ and CD8^+^ T cell responses. Notably, CD4^+^ T cells are less explored than CD8^+^ T cells in relation with influenza-associated immunity. Investigations on post-vaccination-specific immune responses on memory CD4^+^ T cells showed that elderly populations have a higher frequency of specific central memory CD4^+^ T cells (CD4^+^CCR7^+^CD45RA^−^), producing IFN-γ and TNF-α, which could contribute to long-term responses against influenza vaccine [6]. However, compared with young populations, elderly populations have a higher frequency of effector memory CD4^+^ T cells (CD4^+^CCR7^−^CD45RA^−^) and reduced levels of serum IL-7 [6]. These factors might contribute to the failure in expanding anti-influenza responses in elderly populations.

Memory T cells bear a great significance in relation to antiviral vaccination. More specifically, studies have shown that T cell resident memory (Trm) is vital in the prompt killing of virus-infected cells [7]. Trm are derived from immune infiltrates during the effector phase of immune responses and comprise both CD4^+^ and CD8^+^ T cells, and are involved in protective immunity and clearance [8], especially against viral infections [9,10]. Such responses are mostly evident in the early phases of viral infection in which viral replication is limited and immune responses are adequate to prevent disease progression. CD4^+^ Trm in lungs could be derived from Th17 cells and are also involved in rapid action against bacterial infections [11]. The gene profiles of Trm showed high constitutive expressions of cytotoxic mediators and effector molecules. Therefore, the induction of Trm via the local administration of viral vaccines seems to be an attractive approach. However, vaccination based on the induction of influenza virus-specific Trm in lungs is transient and studies have shown that the nasal induction of Trm is more effective at preventing disease progression [3].

A major challenge in developing a universal T cell vaccine for influenza comes in the form of genetic restrictions, which are attributed to diverse MHC alleles (HLA class I) responsible for presenting viral peptides to T cells for recognition. MHC class I molecules present internal viral peptides and are encoded by the HLA-A/B/C genes, while MHC class II molecules present external peptides and are encoded by HLA-DP/DQ/DR genes [12]. These molecules exhibit high polymorphism, which influences peptide binding. T cells typically respond to selected HLA epitopes and, therefore, similar responses are recorded in individuals expressing the same HLA allele. However, epitope recognition by T cells is affected by various factors, such as protein expression and processing and T cell repertoire. HLA alleles allow for the identification of populations with cross-reactive T cells and assess the risk of severity of influenza disease. HLA-A1 allele is considered as an important predictor of influenza-specific CD8^+^ T cells, although HLA-B alleles generate more robust T cell responses [13]. The M1_58–66_ epitope, restricted by HLA-A*0201, is recognized by CD8^+^ T cells from most influenza A viruses [14]. In addition, certain HLA-A alleles have been identified as risk factors for influenza infections due to low peptide binding and have been recorded at varying levels among different population demographics [3]. Therefore, an effective T cell vaccine should comprise of multiple cross-reactive epitope-specific T cell responses, benefitting from the ability of HLA alleles to select highly conserved peptides for presentation.

Successful vaccine development needs to overcome viral immune evasion mechanisms, which impede antiviral immunity. Viruses have been shown to mutate MHC-anchor residues to thwart antigen presentation to T cells. Furthermore, point mutations at the highly conserved anchor residues of influenza A viruses could be the most plausible way for the virus to escape from cytotoxic CD8^+^ T cell surveillance [15]. Moreover, mutations at anchor sites and TCR for influenza epitopes on CD8^+^ T cells have also been observed [16]. Importantly, an engineered influenza virus with a mutation at the viral epitope was able to escape CD8^+^ T cells; however, preemptive exposure to these mutant epitopes resulted in appropriate CD8^+^ T cell responses against the infections with serologically distinct influenza that carried the same mutation [17]. Thus, the tactical priming of T cells against known escape variants could diminish viral immune escape. A vaccine, comprising a wide spectrum of peptides, combining different T cell epitopes and TCR escape variants, could be beneficial in preparing a vaccine for influenza.

Several vaccine candidates are currently being explored to protect against influenza via eliciting favorable T cell-mediated immunity by utilizing replicating and non-replicating viral vectors. These include Modified Vaccinia Ankara (MVA), Simian Adenovirus, adenovirus 5 vectored vaccines and recombinant peptide approaches for the mosaic of conserved peptides or NP with M2e proteins. The delivery of T cell vaccines does not principally require adjuvants, since viral vectors are self-adjuvating. MVA–vectored vaccine expressing NP and M1 (MVA−NP + M1) are capable of producing substantial T-cell responses [18]. Moreover, vaccination with novel recombinant Simian Adenovirus, ChAdOx1 NP+M1 also showed suitable T-cell immunogenicity [19]. The replication-competent Ad4-H5-Vtn vaccine against influenza A H5N1 also exhibited enhanced immunogenicity [20]. Numerous clinical trials are registered for influenza vaccine research, of which approximately 180 clinical trials focus on T cell immunity in relation with influenza vaccines; MV–NP + M1 is explored in nine clinical trials, ChAdOx1 NP + M1 in two, while three clinical trials have been registered for Ad4-H5-Vtn, among other candidate vaccines [21].

Thus, influenza-specific T cell vaccines require better understanding of Trm and viral immune escape, as well as the consideration of HLA alleles and global coverage. Conserved hemagglutinin shows that the development of a universal influenza T cell vaccine could be possible. Moreover, better understanding of T cell responses that correlate with protection against influenza, is warranted. Ultimately, the efficacy of T cell vaccines against conventional antibody-based vaccines would require large-scale comparative studies and the standardization of vaccine production, delivery and testing protocols.

In the current scenario of pandemic threats, the necessity of a novel universal vaccine with broader and greater efficacy than prevailing influenza vaccines against existing strains is inevitable. Numerous approaches, including the inclusion of multiple adjuvants, neuraminidase and improving the immune reactiveness against heterosubtypic strains, are currently under investigation for the development of a factual universal vaccine against influenza [22]. Accumulating evidence suggests that the induction of host T cell responses against internal viral proteins, especially Trm residing in respiratory mucosa, is one of the most promising approaches for productive universal influenza vaccines [3]. Moreover, studies on immunomodulation induced by influenza vaccines could be beneficial for the development of alternative approaches to prevent infections among the immunocompromised and elderly populations. Furthermore, the choice of specific adjuvants and pharmacomodulators, having potential role in priming T cells to elicit favorable antiviral responses, are also important for favorable disease outcome. Further immunological studies are warranted to elucidate heterologous immunity induced by subsequent vaccines and their association with the progression of infection and clinical outcome.
